# Supplementary material for: Pervasive Selection against MicroRNA Target Sites in Human Populations
Source: Mol Biol Evol. 2020 Jun 25;37(12):3399–408. doi: 10.1093/molbev/msaa155 (PMC7743725; doi:10.1093/molbev/msaa155)
Supplement: msaa155_supplementary_data [file msaa155_supplementary_data.zip › Supplementary_Figure_2.pdf]

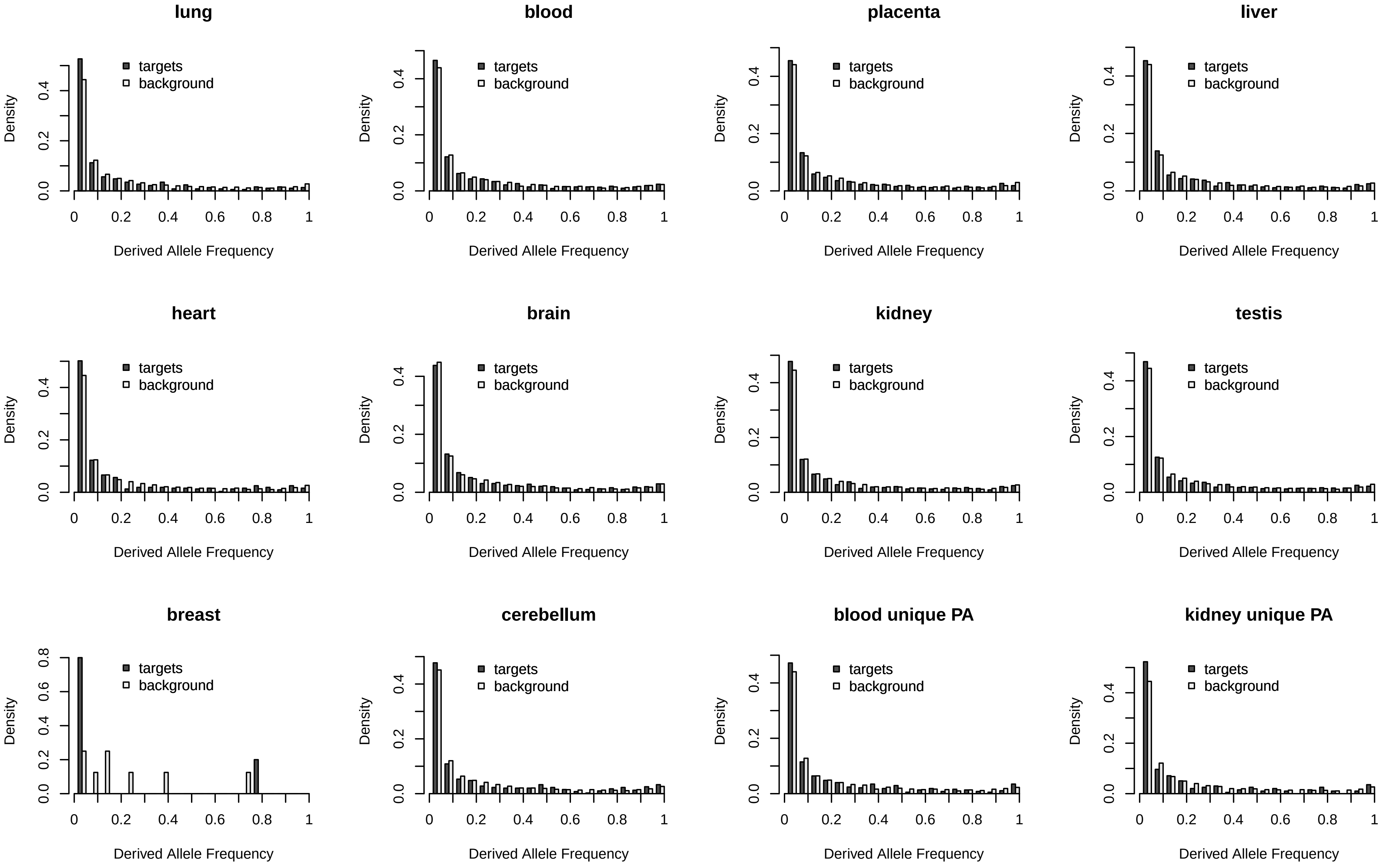
lung
blood
placenta
liver
heart
brain
kidney
testis
breast
cerebellum
blood unique PA
kidney unique PA
targets
background
Density
Derived Allele Frequency
