# Supplementary material for: Pervasive Selection against MicroRNA Target Sites in Human Populations
Source: Mol Biol Evol. 2020 Jun 25;37(12):3399–408. doi: 10.1093/molbev/msaa155 (PMC7743725; doi:10.1093/molbev/msaa155)
Supplement: msaa155_supplementary_data [file msaa155_supplementary_data.zip › Supplementary_Tables.pdf]

Supplementary Table 1. Statistical comparison of the derived (target) allele distributions at potential target sites versus background (expected) for 10 different tissues.

tissue	# SNPs interactions	# SNPs background	p[1]	q[2]
lung	552	2292	0.000	0.001
blood	970	3907	0.003	0.016
placenta	1154	4644	0.006	0.018
liver	932	3803	0.007	0.018
heart	495	1927	0.017	0.028
brain	992	4232	0.026	0.037
kidney	791	3112	0.010	0.021
testis	1126	4552	0.038	0.045
breast	6	36	0.211	0.211
cerebellum	617	2339	0.040	0.045
blood (unique PA)	441	1872	0.027	0.034
kidney (unique PA)	275	998	0.034	0.034

[1] p-value computed from a one-tailed Kolmogorov-Smirnov test. [2] q-value (False Discovery Rate).

Supplementary Table 2. Statistical comparison of the derived (target) allele distributions at potential target sites of highly-expressed microRNAs versus target sites for non-detected (zero expressed) microRNAs for 10 different tissues.

tissue	# SNPs interactions	# SNPs background	p[1]	q[2]
lung	374	6838	0.001	0.005
blood	840	2630	0.027	0.068
placenta	1036	4177	0.196	0.245
liver	726	5488	0.113	0.189
heart	319	7421	0.005	0.023
brain	827	4189	0.688	0.688
kidney	576	7263	0.025	0.068
testis	922	6133	0.133	0.190
breast	5	8	0.061	0.121
cerebellum	396	7602	0.307	0.341
blood (unique PA)	375	2737	0.187	0.187
kidney (unique PA)	197	7455	0.034	0.068

[1] p-value computed from a one-tailed Kolmogorov-Smirnov test. [2] q-value (False Discovery Rate).

Supplementary Table 3. P-values for the statistical support of effect of dependent variables and interaction in two linear models (see main text). P-values below 0.05 are in red.

	Linear model (p-values)		
**Tissue**	**conservation**	**expression**	**interaction**
lung	0.427	0.000	0.334
blood	0.163	0.156	0.262
placenta	0.165	0.182	0.295
liver	0.316	0.038	0.372
heart	0.940	0.020	0.393
brain	0.551	0.640	0.134
kidney	0.694	0.015	0.789
testis	0.945	0.055	0.361
breast	0.830	0.049	0.570
cerebellum	0.900	0.794	0.650
