# Supplementary figures and images for: Pervasive Selection against MicroRNA Target Sites in Human Populations
Source: Mol Biol Evol. 2020 Jun 25;37(12):3399–408. doi: 10.1093/molbev/msaa155 (PMC7743725; doi:10.1093/molbev/msaa155)

**Wobble near-targets**

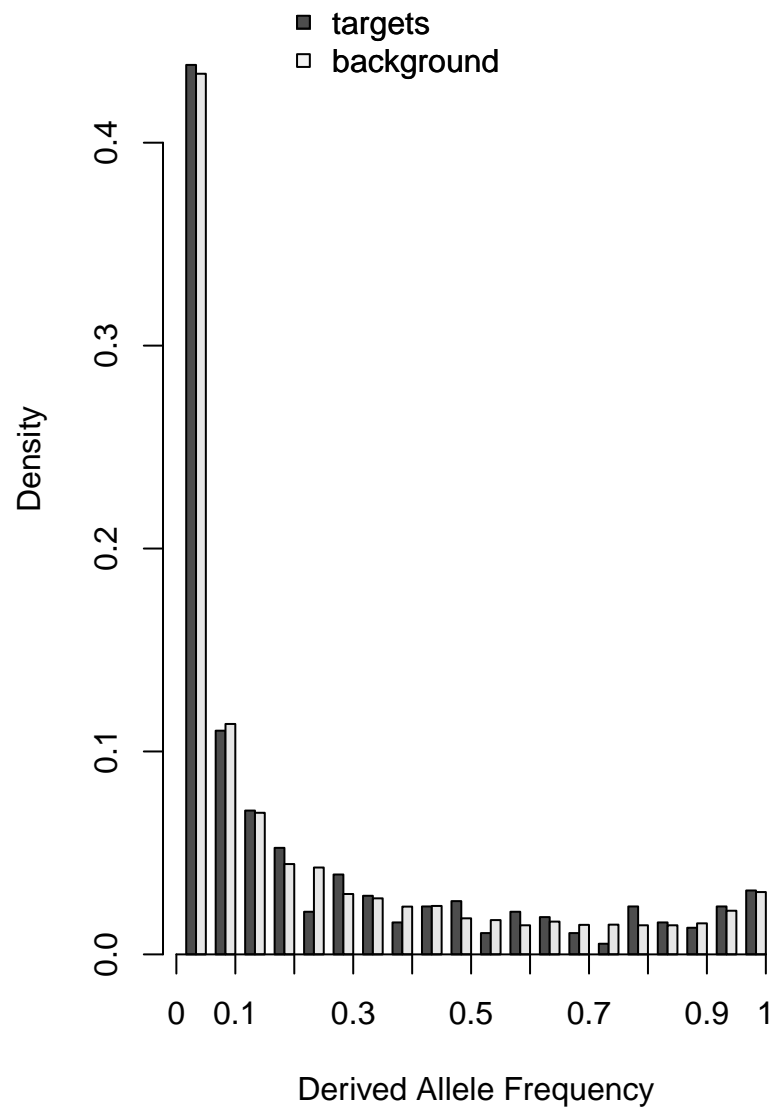

**Non-Wobble near-targets**

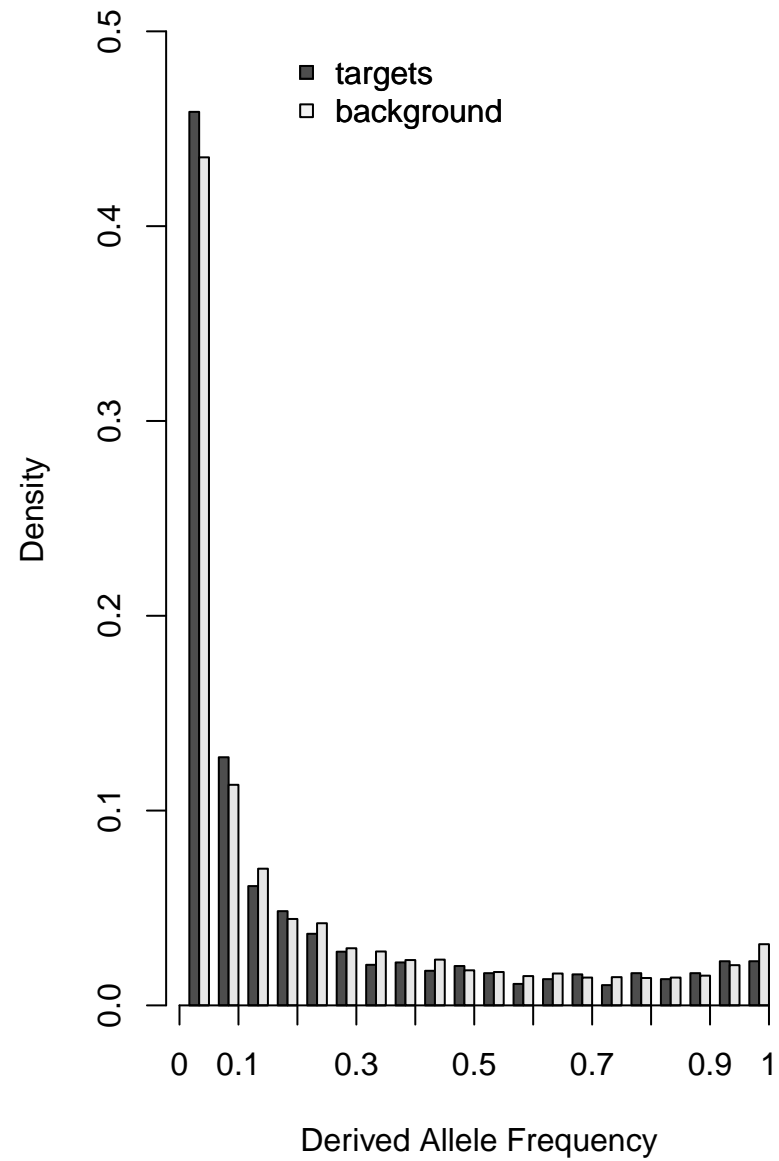

Supplement: msaa155_supplementary_data [file msaa155_supplementary_data.zip › Supplementary_Figure_3.pdf]
